# AlzheimerViT: harnessing lightweight vision transformer architecture for proactive Alzheimer’s screening

**DOI:** 10.3389/fmed.2025.1568312

**Published:** 2025-06-17

**Authors:** C. Kishor Kumar Reddy, Hafsa Ihteshamuddin Ahmed, Muhammad Mohzary, T. Monika Singh, Mohammed Shuaib, Shadab Alam, Hani Mohammed Alnami

**Affiliations:** ^1^Department of Computer Science and Engineering, Stanley College of Engineering and Technology for Women, Hyderabad, India; ^2^Department of Computer Science, College of Engineering and Computer Science, Jazan University, Jazan, Saudi Arabia; ^3^Engineering and Technology Research Center, Jazan University, Jazan, Saudi Arabia

**Keywords:** Alzheimer’s disease, MobileViT, data augmentation, disease detection, deep learning

## Abstract

**Background:**

Alzheimer’s is a disease in the human brain characterized by gradual memory loss, confusion, and alterations in behavior. It is a complex and continuously degenerative disorder of the nervous system, which still has early detection and diagnosis as challenges to overcome. The disease causes significant damage in individuals suffering from the disorder as they progressively lose cognitive ability. Its diagnosis and management depend primarily on the ability to diagnose early to initiate proper intervention. Unfortunately, this remains a difficult feat, given the resemblance of early signs of the disease with symptoms associated with normal aging and other disorders involving cognition. While clinical tests have their limitations, brain imaging such as MRI can provide detailed insights into changes in the brain. Deep learning techniques, mainly when applied to MRI data, have proven helpful in the early detection of Alzheimer’s Disease.

**Methods:**

In the proposed study, a lightweight, self-attention-based vision transformer (ViT) is employed to predict Alzheimer’s disease using MRI images from the OASIS-3 dataset. Data pre-processing and augmentation techniques have been added to strengthen the model’s generalization ability and enhance model performance, which is visualized using Grad-Cam.

**Results:**

The proposed model achieves exceptional results with an accuracy of 98.57%, approximate precision of 98.7%, Recall of about 98.47%, and specificity of 98.67%. It also achieves a Kappa Score of 97.2% and an AUC ROC Score of 99%.

**Conclusion:**

This paper, along with comprehensive data pre-processing and augmentation, represents one of the major steps toward achieving more robust and clinically applicable models for Alzheimer’s disease prediction. The proposed study indicates that deep learning models have the potential to enhance the diagnosis of Alzheimer’s disease. By integrating Deep learning techniques with careful data processing, more reliable early detection models can be developed, which in turn leads to better treatment outcomes.

## 1 Introduction

Alzheimer’s disease (AD) is an irreversible and also progressive neurodegenerative disease that gradually deteriorates the memory, cognitive abilities, and activities of daily living. AD accounts for almost 60 to 80% of cases of dementia. While brain cells gradually degenerate and die, symptoms in an AD patient worsen and include confusion, disorientation, and forgetting. Daily activities are impacted when the person becomes unable to perform simple tasks. This disease has been widely thought to occur in old age; however, the exact etiology is still not clear, with a possible interaction of genetic predisposition, environment, and lifestyle factors thought to act causally on the disease. Dementia generally is considered to refer to a syndrome involving a group of progressive memory disorders and difficulties thinking and daily functioning. Older persons are said to have been the most populous part of individual cases of dementia. In contrast, it is noted that younger-onset dementia makes up an even smaller proportion of people with dementia. Injury or disease causes brain damage. Alzheimer’s disease is mainly the cause of more than half of dementia in 60–70 percent of cases (1). Alzheimer’s disease is the first category of dementia. It involves brain diseases that produce different effects, such as loss of memory affecting cognition, problems with behavior along with memory being affected, and so forth (2). The other degrees of dementia are vascular dementia, Lewy bodies dementia, and frontotemporal dementia. Symptoms of dementia, such as memory loss, confusion, problems with problem-solving, mood or behavioral changes, etc., become more severe with time (3). The main risk factors are the following: advanced age, hypertension, diabetes, obesity, smoking, heavy drinking, depression and social isolation, lousy nutrition, and low physical or cognitive activity (4).

Although there is still no cure, proactive measures can enhance the quality of life among dementia patients and, by extension, their family caregivers. Keeping cognitions intact requires physical and social activities, treatment of other health conditions, and mental activity. Caregiver emotional and physical exhaustion is most often viewed as the prime reason for people seeking to institutionalize people with dementia. It makes it very important for a caregiver to find support from family and professionals, as well as engage in stress management programs. Advocacy is itself necessary since most people here are suffering from stigma, poor care, and rights violations arising from physical and chemical restraints in care centers. A rights-based legislative framework is required to dignify high-quality care for the lives of the people who have dementia and their caregivers. There is awareness, early intervention, and community involvement. People with dementia can do things to be active and involved and keep their brains stimulated, which will help even maintain their daily functioning and improve their quality of life (5). Provide physical, participatory activity, social interaction, or environmental stimulation to maintain daily functioning. Caring for and supporting a person living with dementia has its challenges, which really affect the health and well-being of the carer. As a supporter of the person living with dementia, this means reaching out to family and friends as well as professionals for support and making time to take care of oneself on a regular basis. Try some stress management techniques like mindfulness-focused exercises and seek professional help and advice if necessary. Diagnosis of dementia and determination of the subtype is a long and complicated process involving many parts of information pooled together from different sources like clinical history. The 2023–2024 World Health Organization data (6) highlights country-specific mortality rates per 100,000 for Alzheimer’s disease, differentiated by gender. Supplmementary Figure S1 illustrates a comparative bar graph analysis, shedding light on the varying impacts of this widespread condition across populations.

This research has been motivated by the great need for better early detection and diagnosis of dementia, a progressive neurological illness that plagues millions worldwide. Alzheimer’s Disease (AD) and other types of dementia are particularly troublesome because the methods of diagnosis often depend on clinical behavior, which manifests only in the later stages of the disease. Effectively detecting the condition early is essential to its remedial interventions and to delaying its progression; established methods fail to identify subtle changes in the brain, which would precede these overt symptoms.

In populous countries like the United States (1), approximately 4.5 million individuals are currently living with Alzheimer’s disease, with this figure projected to rise to 14 million by 2050. Various machine learning techniques and deep learning approaches have been employed for dementia classification and prediction using datasets like OASIS and ADNI. Deep learning models such as DenseNet201, MobileNet, VGG19, and ResNet152 achieved accuracies ranging from 90 to 93%, though limited by dataset diversity and overfitting concerns (7). Logistic regression with cognitive test predictors from ADNI data yielded an 85.8% positive predictive value but lacked long-term progression tracking (3). Gradient boosting models, such as Ensemble Gradient Boost, reached 91.2% accuracy but suffered from small sample sizes and limited clinical relevance (4). Basic Machine Learning models, including Gaussian Naïve Bayes and SVM, achieved high accuracy (95%) with Cuckoo optimization but were constrained by dataset diversity (5). Previous studies using models like Naive Bayes and MLP demonstrated promising F-measures but encountered challenges with generalization (6). Deep reinforcement learning balanced MRI classes with 90.23% accuracy, but small sample sizes restricted real-world applicability (8). Supervised learning with convolutional neural networks from ADNI achieved accuracies of up to 93% but lacked clinical validation (7). Questionnaire-based studies, while simpler, recorded moderate accuracy (0.81) but were biased due to self-reported data (9).

The limitations of machine learning algorithms are particularly significant in healthcare, where reliability, transparency, and trust are critical. A major concern is the lack of explainability, as many ML models function as “black boxes,” offering little intuition into their decision-making processes, which makes it difficult for healthcare professionals to trust and apply them. Many studies (2, 3) rely on small datasets, such as OASIS, ADNI, or limited MRI sessions, which restrict generalizability and increase the risk of overfitting, while a lack of clinical validation further limits their real-world applicability. Dataset biases and limited diversity, particularly in studies using questionnaire-based data, hinder broader applicability to diverse populations (8). Overfitting remains a challenge, especially in complex models like Ensemble Gradient Boosting or Gray Wolf Optimization-based EDCM (7), and reliance on self-reported data introduces biases that reduce reliability (8). Short-term data constraints further impede the ability to predict long-term dementia progression, while the complexity of these models often affects their interpretability and practical deployment. However, traditional machine learning methods have struggled with the complexities of AD detection (10–12). Identifying specific features within relevant brain image patterns is essential but remains a difficult task.

Additionally, ML methods require large, high-quality labeled datasets, which are tough to obtain due to privacy concerns, annotation costs, and the need for domain-specific expertise. The computational resources needed to train and optimize complex models can be prohibitive in resource-constrained environments. In this paper, we aim to address these issues by moving beyond traditional ML/DL methods for early AD detection based on handwriting, focusing on enhancing explainability to provide healthcare professionals with clear, interpretable insights, fostering trust, and improving clinical applicability.

The current study introduces AlzheimersViT, a hybrid architecture designed to detect subtle structural changes in the brain as early indicators of dementia by combining the strengths of Convolutional Neural Networks (CNNs) and Vision Transformers (ViTs). AlzheimersViT leverages MobileNet V2’s efficient inverted residual blocks and depth-wise separable convolutions, which optimize computational performance while retaining complex feature representations. These are complemented by Vision Transformers, which capture long-range dependencies and global context, enhancing the model’s ability to recognize fine-grained patterns in brain imaging data. The architecture incorporates linear bottleneck layers and reversible residual connections to mitigate the vanishing gradient problem and improve training stability in deep networks. Additionally, it offers adaptability through hyperparameters like width and resolution multipliers, enabling customization for resource-constrained environments. This combination of efficiency, flexibility, and accuracy makes AlzheimersViT a powerful tool for early-stage dementia detection, with significant potential for real-time applications on mobile and embedded platforms.

Major Contributions of the proposed AlzheimerViT are:

i The proposed system employs dataset augmentation and feature engineering methods on the OASIS-3 dataset to support performance and generalization. Techniques used for data augmentation include rotation, flipping, shearing, and grayscale, hue, saturation, and brightness adjustments to bolster the model’s resilience to variability in images.
ii The proposed system, AlzheimerViT, uses a lightweight Vision Transformer to predict Alzheimer’s disease from MRI images, demonstrating the feasibility and effectiveness of modern neural networks in medical diagnostics.
iii AlzheimerViT was evaluated and compared with other models such as ICAE (Transfer Learning), ICAE, Ensemble with feature selection (AD-MCI), SVM with RBF, CAE, Ensemble with feature selection (MCI-CT), Ensemble with feature selection (AD-CT), Multilayer Perceptron, LIBS-ML, and qEEG Processing Technique. The results clearly show that the proposed model outperforms the existing models.

The remaining paper is structured as follows: Section 2 provides an overview of the related work, Section 3 details the materials and methods, Section 4 represents the results and analyzes them in comparison with existing approaches, and Section 5 concludes the study. References are included at the end.

## 2 Background

Significant advances on the Alzheimer’s disease (AD) front have recently been made in terms of early detection and treatment development. By the year 2024 alone, more than 170 clinical trials have emerged directing disease-modifying therapies aimed at treating the underlying mechanisms of AD; a significant number of these, that focus on amyloid-targeting treatments, have shown promise in the mitigation of cognitive decline, especially at early phases of the disease. The FDA is expected to decide on approval regarding donanemab, an amyloid-clearing drug that has been shown to slow AD progression. Deep learning technologies, such as DenseNet201, MobileViT, VGG19, and ResNet152, have been implemented to classify dementia in different stages using MRI data from the OASIS dataset. The outcomes of these studies indicate a good accuracy-93% of DenseNet201, MobileViT, and VGG19, and 90% of ResNet152; however, the generalizability of these results remains limited because they may be biased and do not cover diverse populations or settings (7). Other works that employed machine learning models like logistic regression and ensemble gradient boosting have also yielded very high accuracies but suffer from overfitting very small sample sizes and lack of clinical verification (9).

Several other research efforts have implemented various machine learning algorithms, like Light Gradient Boosting, XGBoost, and support vector machines (SVM), to predict the risk of dementia. One of the studies cites the use of some datasets from ADNI to arrive at an 85.8% sensitivity value for positive predictive value and 92.2% for negative predictive value. Still, this trend should have been more generalizable to a broader audience due to the restrictive diversity of populations in the sample (10, 11) adds that using the Gaussian Naïve Bayes and SVM methods, the achieved level of accuracy is 95%, of precision 97%, and Recall is 95%. However, there is limited diversity in the OASIS dataset, and the models used are simple. In contrast, more novel yet still complex approaches, such as those using deep reinforcement learning-based models, also give promising results. Still, small sample sizes and the risk of overfitting continue to hamper progress in translating these models to real-world healthcare applications. In a study that implemented CNNs for dementia classification, normal cognition versus early mild cognitive impairment (EMCI) was classified at 92.5%, and Alzheimer’s detection at 90.5% accuracy. Although these models were quite successful, they also demonstrated some shortcomings in clinical validation and real-world applicability (12).

Clinical questionnaires and MRI session data have long served as key datasets for dementia detection by machine learning models. One such model, which relied on a 37-item questionnaire completed by a sample size of 5,272, achieved a diagnosis accuracy of 0.81. Note that the model lacks clinical validation and relies on self-reported data, which may introduce bias into any results derived from it (13). MRI-based approaches also included EDCM, the Enhanced Dementia Detection and Classification Model, which utilized Gray Wolf Optimization for the feature selection and later showed improved accuracy after optimization. However, the study sample is said to be so low that the results will suffer for this reason and to the risk of overfitting due to model complexity (14). Despite this, another study using machine learning based on 285 subjects into two small subgroups achieved an admirable 96% success in dementia detection. Still, concerns over small sample size and model complexity created questions about overfitting (15). Furthermore, many approaches, such as decision tree and random forest, have been applied to AD detection, but most of these approaches strongly depend on high-quality training data. Privacy-related challenges have emerged, including fears of using patients’ medical records (16).

Numerous deep learning and machine learning studies show promise for application in AD detection but come with a handful of limitations. Studies have demonstrated that efficiency in results has been improved, especially with deep learning models. Still, such approaches are often hampered by many challenges, which include time-horizon data dependency and overfitting. The generalization of findings into global populations is blocked by the clinical dataset with no diversity, such as an ADNI. Methods, amyloid, and tau-tangle imaging have also been explored but with minimal applications, especially in resource-poor settings (17). Despite the clinical efficacy deep learning has shown, there are still problems, such as inconsistent image acquisition and biases in data sets. Examples of these include using MRI imaging for AD detection (18). Compared to previous experiments, the 2021 longitudinal MRI dataset included 5-fold cross-validation on OASIS, which improved the model’s robustness. However, it faced complications due to missing SES and MMSE values, which may have introduced biases, limiting the model’s representativeness (19). Deep Neural Networks were some of the approaches applied for detecting AD through the OASIS dataset, which had no significant shortcomings. However, the study pointed out the need for comprehensive documentation to ensure replicate ability and scalability (20). A further research study about neural networks used the ADNI dataset, obtaining encouraging results but still contending with challenges like overfitting and high computation expenses (21). Moreover, a CNN-based method with MRI data from ADNI and SNBUH indicated inconsistencies because different MRI machines (Philips vs. Siemens/GE scanners) were adopted (22). Finally, various methods, such as the support vector classifiers (SVC), need to be investigated for AD detection using the ADNI dataset; however, they all have shortcomings like overfitting and the need for huge, diverse datasets to ensure broader applicability. While these studies provide a good start, the evidence suggests that better diversity needs to be achieved in datasets, that models need to be generalized, and that clinically validated approaches for Alzheimer’s detection should evolve (23). They focused their work mainly on sparse brain images and did not focus on the global brain patterns that are important for better predictions. Table 1 specifies a thorough analysis of the existing relevant work, along with the models, performance metrics, and demerits.

**Table 1:** Summary of recent AD prediction.

Ref No.	Model name	Dataset	Performance metrics	Demerits
(7)	Random Forest	OASIS-2	Accuracy: 95.53%	Less interpretable; sensitive to feature selection methods.
(9)	Logistic regression model	Alzheimer’s Disease Neuroimaging Initiative (ADNI) dataset BioFINDER 1 dataset	Accuracy: 89.0% Specificity: 86.9% Sensitivity: 91.5% Positive predictive value: 85.8%; Negative predictive value: 92.2%.	Limited diversity of samples will indeed greatly influence generalizability to a broader population. The adequacy of the new unseen data decreases due to the possibility of overfitting.
(10)	Light Gradient Boost.	373 individual imaging sessions from patients. -Minimum of two visits with one-year gaps.	The model achieved 91.2% accuracy.	373 sessions may not be encompassing the entire dementia population. It lacks clinical validation, rendering it less relevant in practice than in healthcare. The level of complexity raises questions regarding overfitting and insufficient information on how it has been addressed.
(11)	Support Vector Machine	OASIS dataset used for dementia prediction. Open access series of imaging studies dataset.	Accuracy: 95%	Factors affecting model performance
(12)	Naive Bayes	Gangbuk-Gu Dementia Screening and CERAD-K Test Dataset (2008–2013, Korea)	Accuracy:71.44% Precision: 0.713% Recall: 0.712% F-measure: 0.712%	Limited generalizability due to the small dataset size.
(13)	DRL-XGBOOST (Deep Reinforcement Learning)	Series of Imaging Studies (OASIS) dataset.	Accuracy: 84.34% Precision: 83.45% Recall: 82.12% F-score: 80.23%	Small sample size in the OASIS dataset may limit generalizability.
(16)	Decision Tree with Ensemble Learning	OASIS-3 Dataset	Accuracy: 87.2%, Precision: 86.1%, Recall: 84.9%	Model sensitivity to imbalanced datasets
(28)	Naive Bayes classifier combined with feature selection techniques. A 37-item questionnaire was filled out by 5,272 individuals.	Show Chwan Health System Register-Based Dataset (IRB 1041208)	Accuracy: 0.81% Precision: 0.82% Recall: 0.81%, F-measure: 0.81%	Limited to questionnaire data, no clinical validation, and possible bias from the self-reported nature of the dataset.
(29)	Extra Tree Classifier	Younger Onset Dementia Dataset (Healthdirect Australia) Dementia Classification Dataset (Kaggle)	Accuracy: 85%.	Dataset Size and Diversity Computational Complexity Feature Dependence
(30)	Edge-Preservation Coherence Improvement (EP-CI) algorithm for image enhancement. The Efficient Fuzzy C Means Adaptive Thresholding (EFCMAT) algorithm -2D-Adaptive Consensual Filter (2D-ACF)	BraTS 2018: Brain Tumor MRI Segmentation Dataset	Accuracy:82%	Focus Only on ROI Segmentation No Validation on Real-World Datasets Lack of Comparative Analysis
(31)	LSTM with Attention Mechanism- Explainable Machine Learning Workflow (using SHAP for interpretability)	NACC and ADNI datasets	Accuracy: 88.6%, Precision: 89.4%, Recall: 87.2%	High computational cost, requires large-scale validation
(32)	Gradient Boosted Machines (GBM) and ResNet-50	Alzheimer’s Disease Neuroimaging Initiative (ADNI) dataset	Accuracy: 91.5%, AUC: 0.94%	Requires extensive computational resources; potential overfitting in ResNet-50; limited interpretability of GBM
(33)	XGBoost Model	MRI Images dataset (specific dataset not mentioned)	Accuracy: 92.3%, Sensitivity: 90.1%, Specificity: 93.8%	Limited dataset diversity; potential for overfitting; lacks interpretability in clinical settings
(34)	Support Vector Machine (SVM), Relevance Vector Machine (RVM)	Structural MRI data from ADNI	Accuracy: 80–85% (SVM), 82–88% (RVM)	Limited sample size; potential overfitting; generalizability concerns
(35)	SVM	ADNI dataset	Accuracy: 75%	Low model performance

## 3 Materials and methods

### 3.1 AlzheimerViT architecture

**Figure 1:**
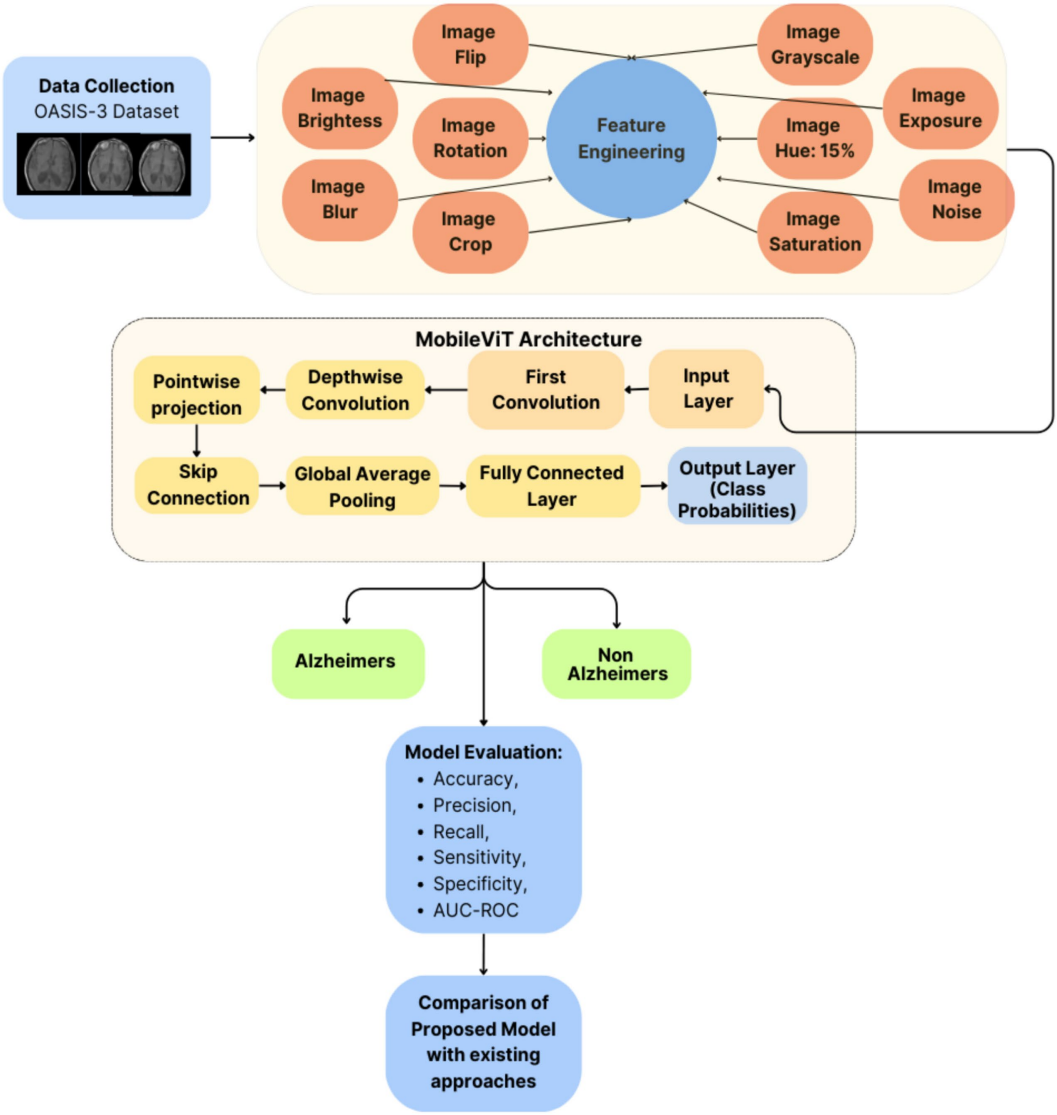
AlzheimerViT architecture.

Figure 1 depicts the proposed Model for Alzheimer’s disease prediction. It portrays the architecture of the AlzheimerViT model, which consists of the following main components: Data Collection, Training Data Pre-processing and Augmentation, and the AlzheimerViT model for Feature Extraction and Classification.

### 3.2 Data collection

**Figure 2:**
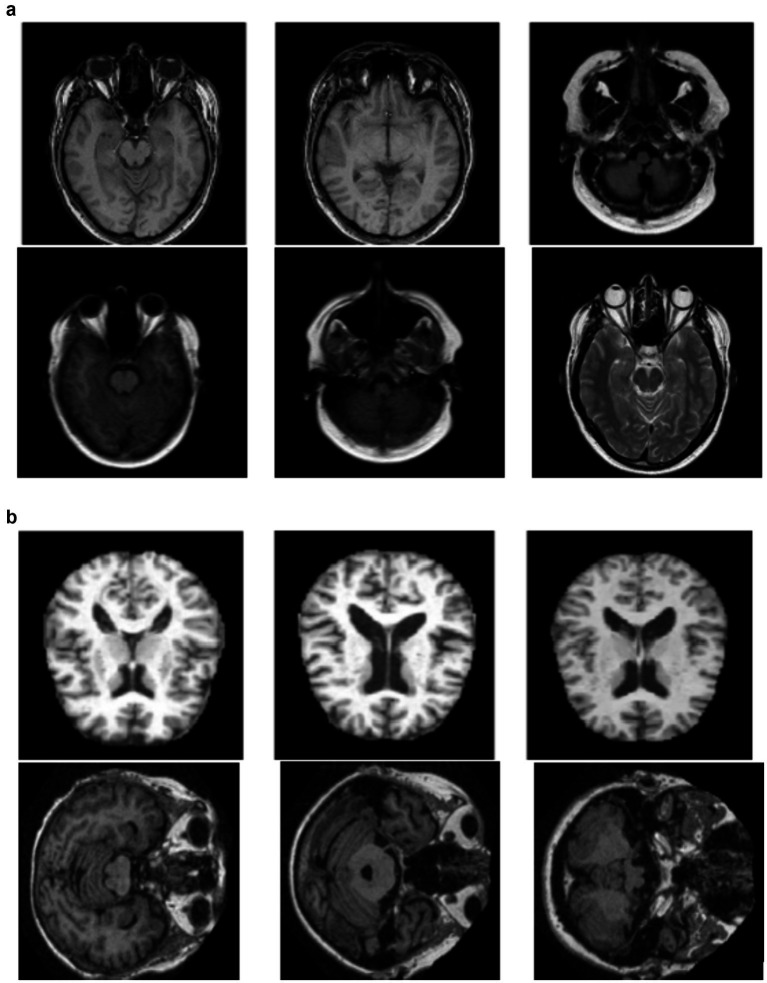
**(a)** Sample images indicating a non demented Brain MRI scan. **(b)** Sample images indicating a demented brain-MRI scan.

The OASIS-3 dataset is a very large and multi-modal dataset, purposed to support research in aging, Alzheimer’s disease, and their comorbidities out of which Axial Brain view of the OASIS-3 dataset was utilized for experimentation. It contains information from more than 1,000 participants, with over 3,000 imaging sessions from structural MRI scans associated with clinical assessments, test scores for neuropsychology, and demographic information for control, as well as participants of mild cognitive impairment and Alzheimer’s disease. Figures 2a,b depicts 6 sample images of non demented and demented brain MRI’s taken from the Oasis-3 dataset. The MRI scans are T1-weighted, high-resolution images that can be analysed visually or quantitatively to investigate brain structure, while clinical data allow the study of cognitive decline and its relationship with neuroimaging features. OASIS-3 is designed to enable many research applications, such as machine learning and predictive modelling of early Alzheimer’s disease.

### 3.3 Training data pre-processing and augmentation

**Figure 3:**
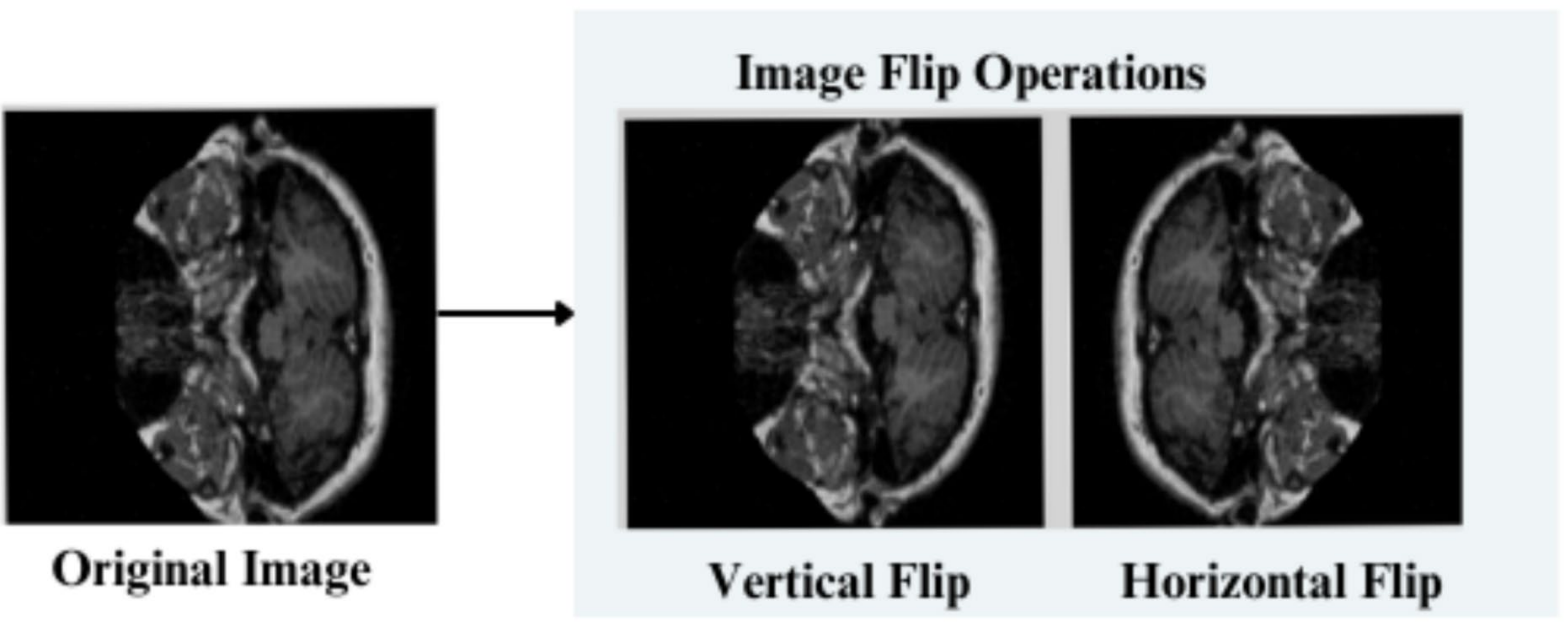
Feature engineering-image flip operation.

**Figure 4:**
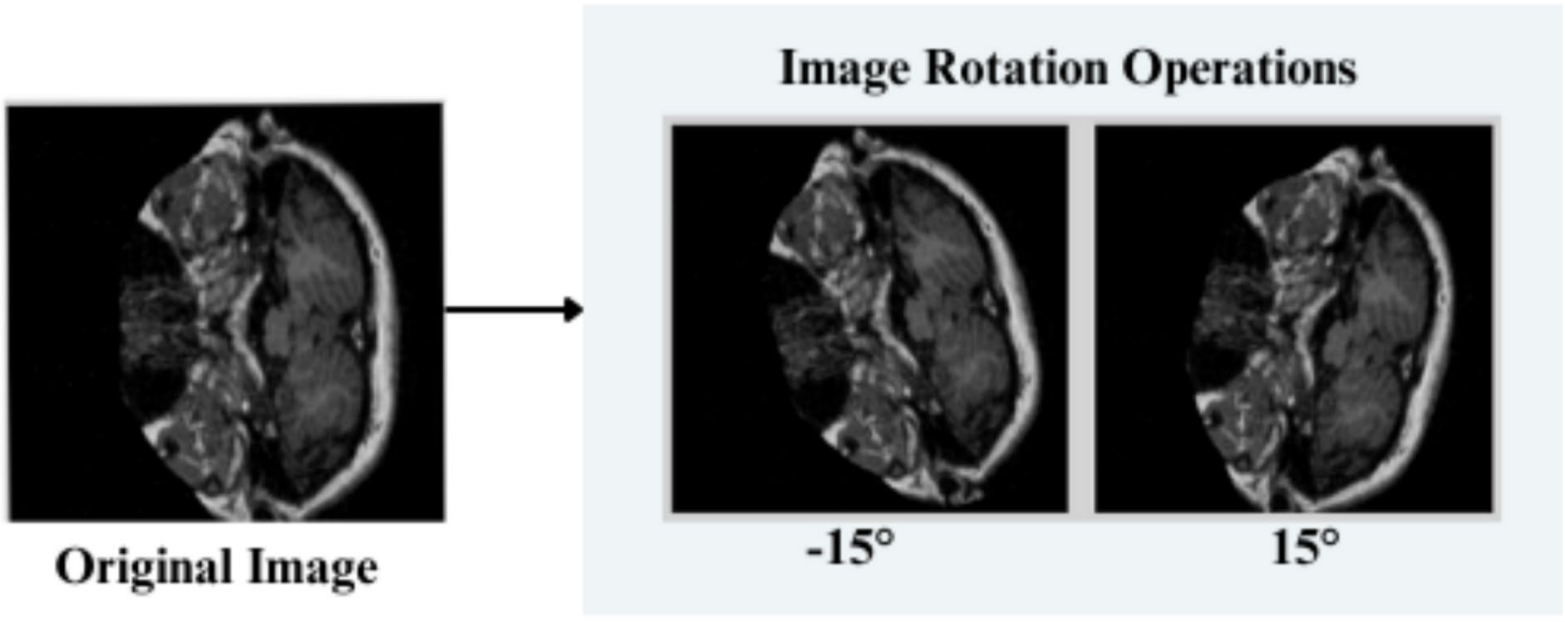
Feature engineering—image rotation operation.

**Figure 5:**
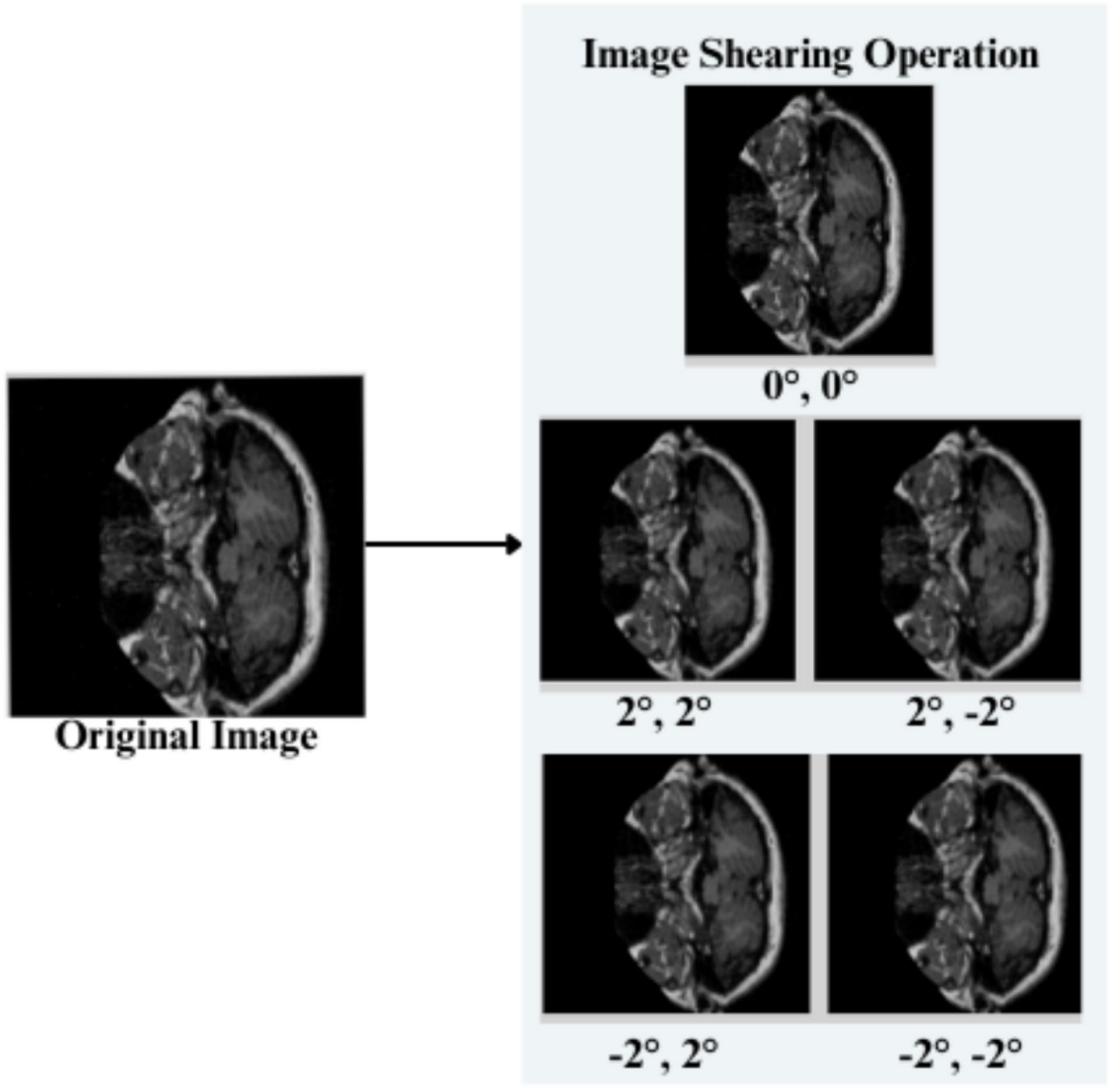
Feature engineering—image shearing operation.

**Figure 6:**
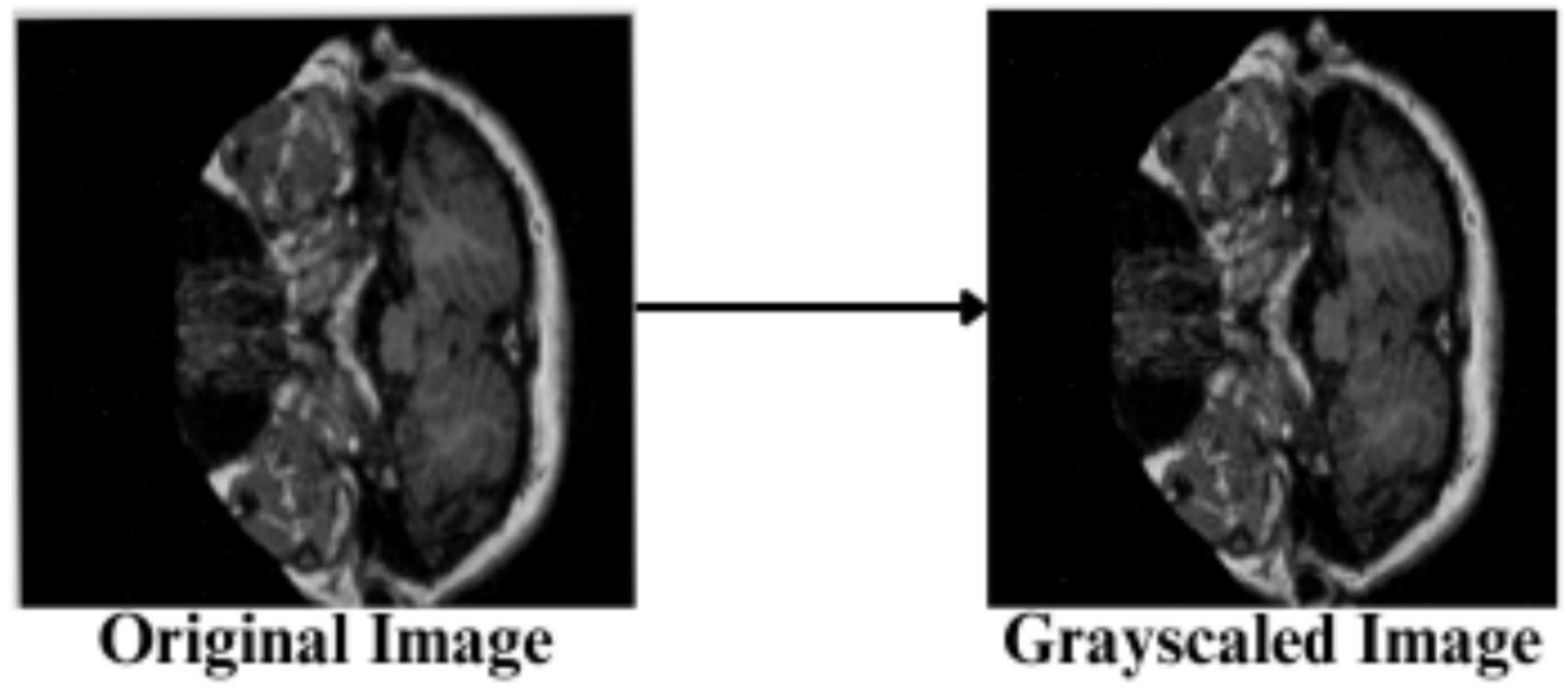
Feature engineering—grayscaling of image.

**Figure 7:**
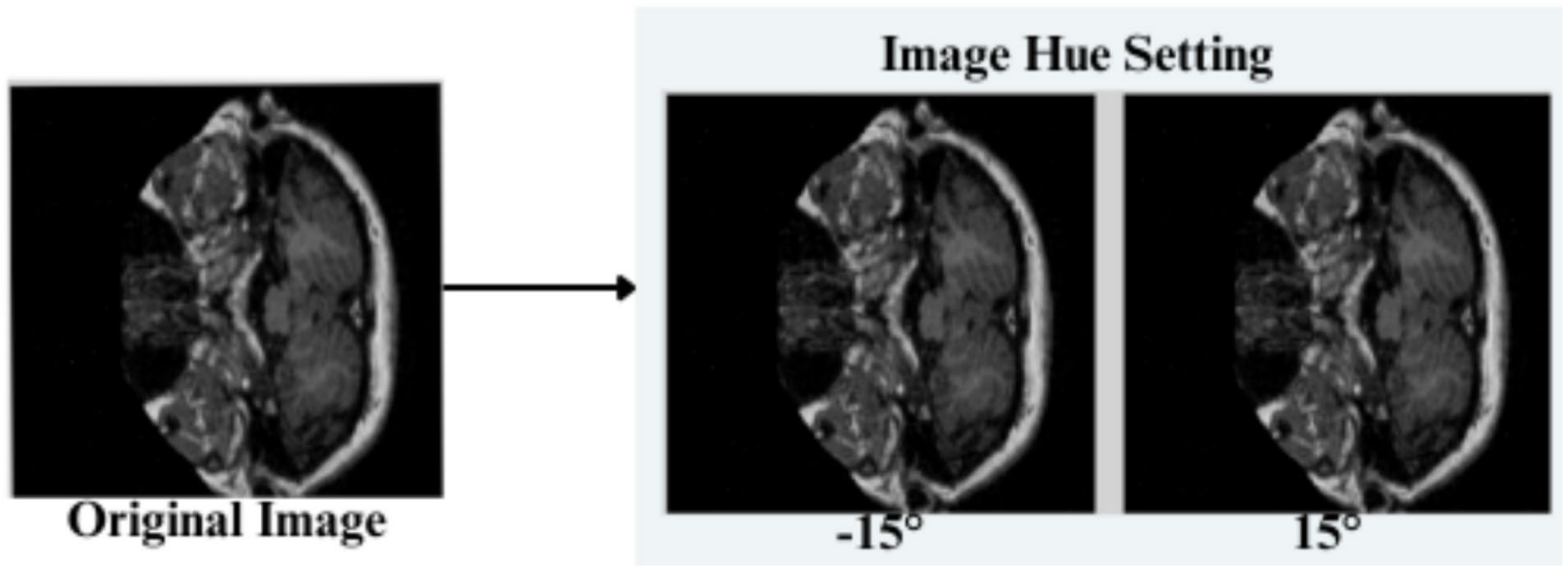
Feature engineering—hue setting of image.

**Figure 8:**
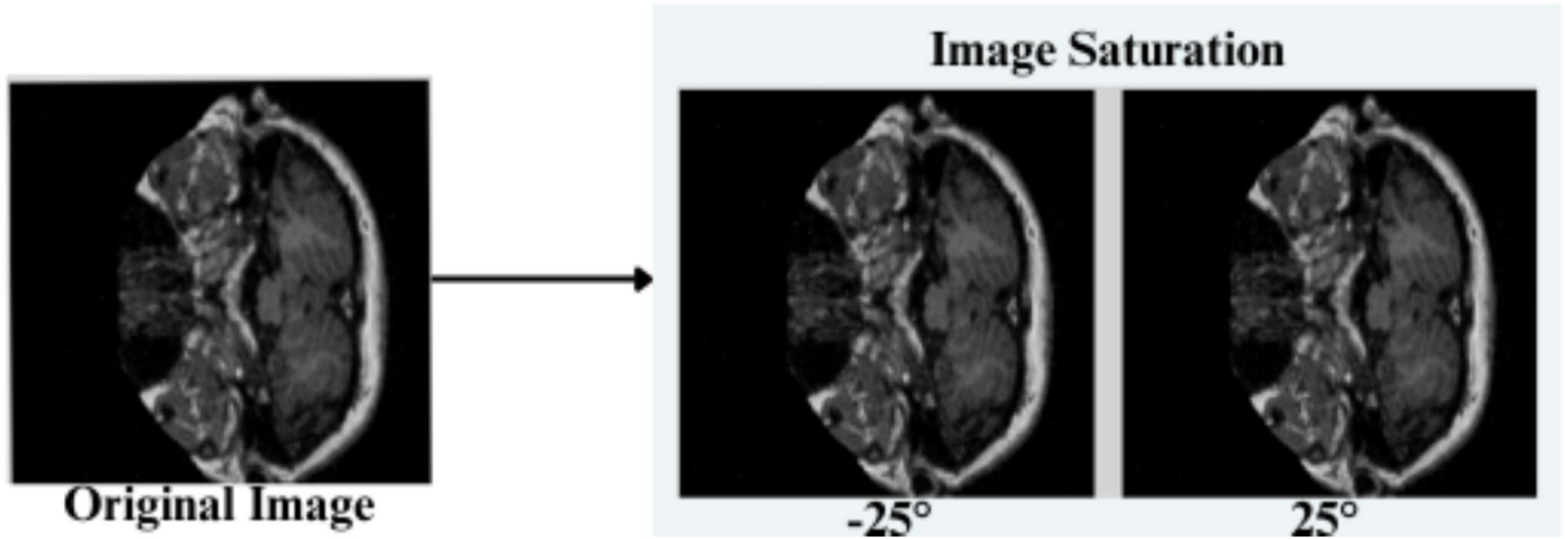
Feature engineering—saturation of image.

**Figure 9:**
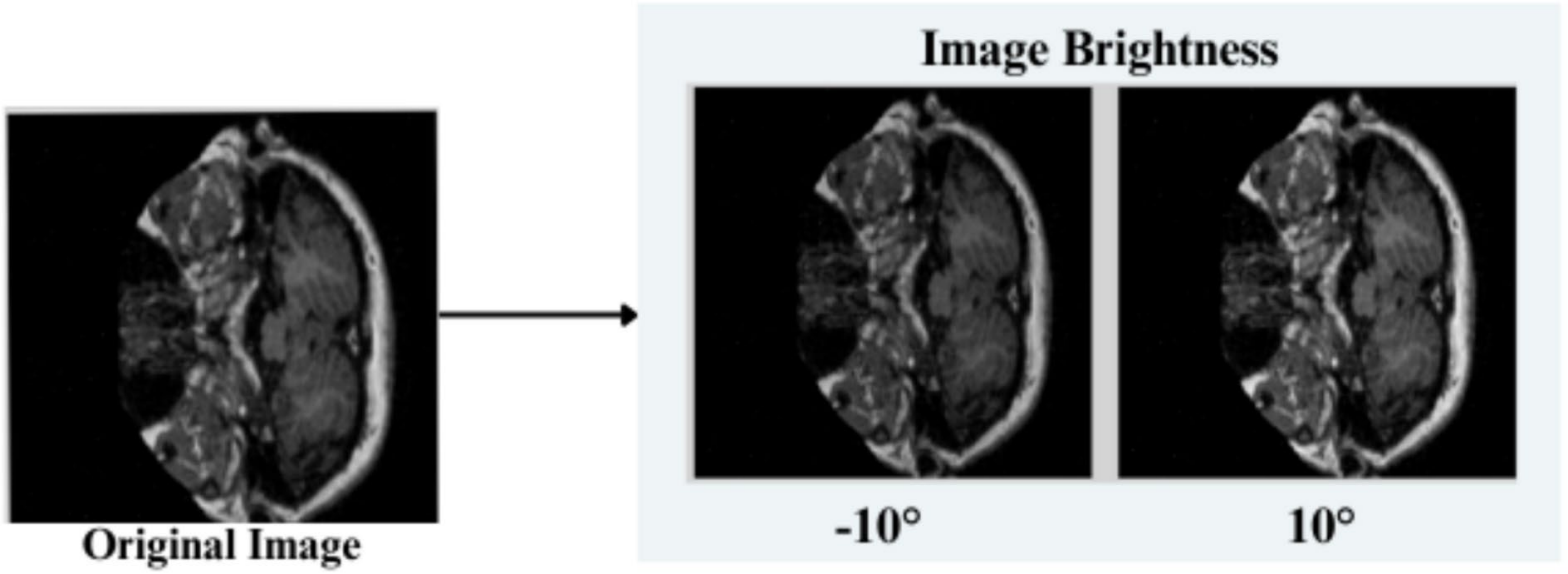
Feature engineering—image brightness setting.

The feature engineering process for this study is to process and augment MRI image data so that relevant patterns associated with Alzheimer’s Disease can be captured. All images are resized to 256 × 256 pixels so that input size is standardized for neural networks, and the same is done to ensure all images have uniform dimensions in order to train the model. It reduces computational complexity, making the processing faster. The next step makes use of image augmentation techniques with the training set, thus artificially increasing the model’s capabilities to generalize new yet unseen data. These techniques include brightness alterations, horizontal and vertical flips, zoom, rotation, and shearing. Horizontal and vertical flipping was performed with 50% chance, and rotation was done clockwise, counter-clockwise, and upside down at random. Cropping was used with a zoom from 0% to a maximum of 4%. Rotation in the −1° to +1° range was used, and shearing transformations were used with ±2° shears in the horizontal and vertical directions. 10% of the images were processed by grayscale conversion and hue adjustment ranging from −15° to +15°, saturation from −25 to +25%, and brightness ranging from −10 to +10%. Those transformations are able to replicate somewhat real-world variations or imperfections that might take place in MRI scans, like different patient positions, variations in lighting, and some variations in the quality of the scan. These techniques help it learn to recognize the patterns related to disease under different conditions and be more robust to variations in the data. Figures 3–9 visually depict the feature engineering operations utilized in this study.

#### 3.3.1 Image flip

Horizontal flip (h)- Pixels at a location (x,y) are transformed as in Equation 1


(1) h(x,y)=(w−x,y)

Vertical Flip (v): Each pixel (x,y) is transformed as shown in Equation 2


(2) v(x,y)=(x,h−y)

#### 3.3.2 Image rotation

*Clockwise Rotation:* Input images are rotated in a clockwise direction, and the resulting coordinates (x′,y′) for each of the pixels is given as shown in Equation 3


(3) x′=y;y′=W−x

*Counter-Clockwise Rotation:*Input images are rotated in a clockwise direction. The resulting coordinates (x′,y′) are explained in Equation 4


(4) x′=H−y;y′=x

#### 3.3.3 Image shearing

A random factor in reference to the study is selected, i.e., Sx, Sy,to shear the input image horizontally and vertically. The transformed pixel coordinates are given by Equation 5


(5) $$x\prime =x+S_{y}.y;y\prime =y+S_{x}.x\;$$

#### 3.3.4 Image gray scaling

10% of the input images are converted to the Grayscale range, shown in Equation 6, utilizing the RGB values combined into a single luminance.


(6) $$\;P_{\text{grayscale}}=0.2989.P_{R}+0.5780.P_{G}+0.1140.P_{B}$$

#### 3.3.5 Image hue adjustment

The input image’s Hue is adjusted by a random value ranging between −15 to +15%.

#### 3.3.6 Saturation adjustment

Input image pixels are readjusted by a random value in a range −25 to +25%, as shown in Equation 7


(7) $$P_{\mathrm{new}}=p*(1+\text{saturation}\_\text{factor})\;$$

#### 3.3.7 Image brightness

The brightness of the input images is readjusted in the range [0.90, 1.10] as explained in Equation 8


(8) $$\;p_{\mathrm{new}}=p*\text{brightness}\_\text{factor}$$

**Figure 10:**
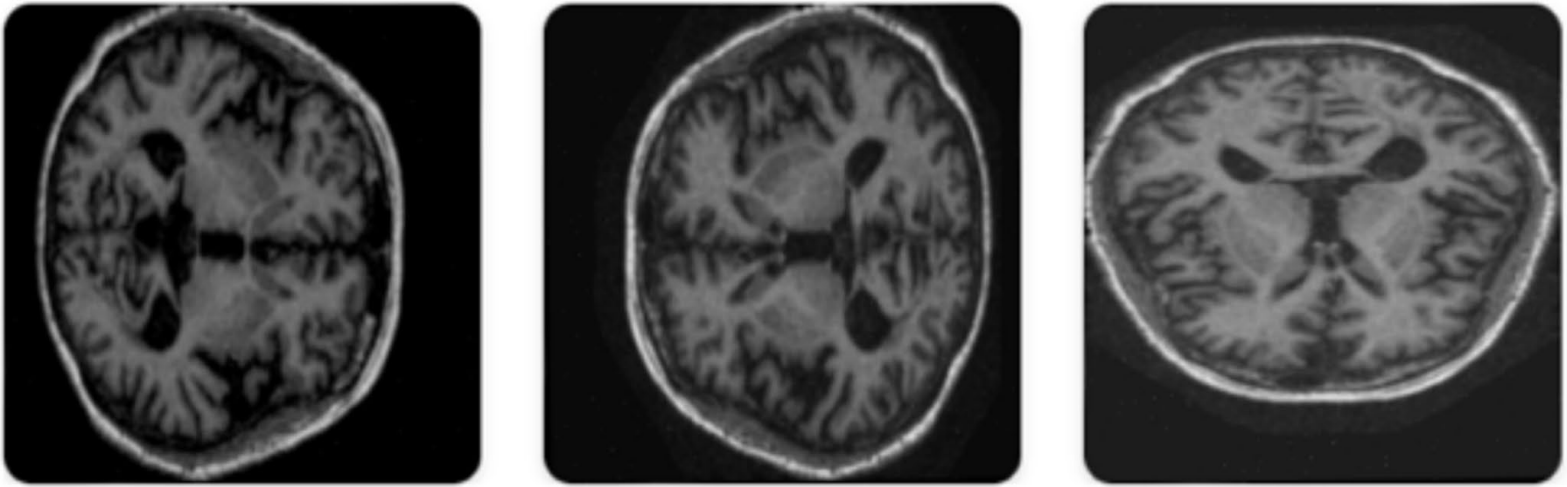
After pre-processing.

**Figure 11:**
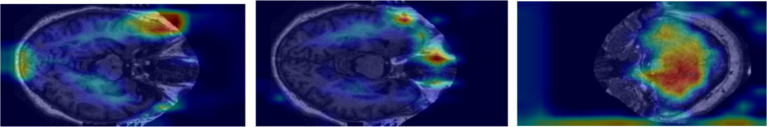
Heatmaps indicating Alzheimer’s disease.

**Figure 12:**
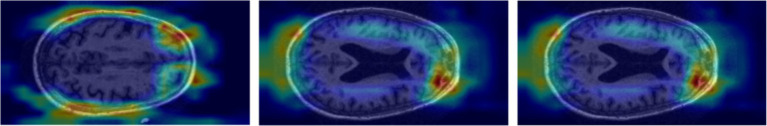
Heatmaps indicating non-Alzheimer’s disease.

For the pre-processed input images shown in Figure 10, the Grad-CAM method is used to produce heatmaps of important regions for classification. Figures 11, 12 are the Grad-CAM heatmaps for Alzheimer’s and non-Alzheimer’s cases, which highlight different regions of the brain indicative of the presence and absence of the disease.

### 3.4 AlzheimerViT feature extraction and classification architecture

AlzheimerViT is a MobileViT architecture that merges the strengths of both CNNs and transformers to effectively capture both local and global dependencies of image data for predicting Alzheimer’s disease (24). Although CNNs (25) are efficient at capturing local features via convolutional operations, they suffer from limited abilities to work on the long-range dependencies in a local area. Transformers (26) are, instead, very effective in learning the global relationship between objects but require a large computational cost and often lack spatial inductive bias. MobileViT addresses this challenge by relying on CNNs for local feature extraction and using Transformers (27) in a lightweight fashion for global context modeling. With this combination, MobileViT can perform well on visual tasks in a computationally efficient manner that is suitable for mobile and edge devices with limited resources.

**Figure 13:**
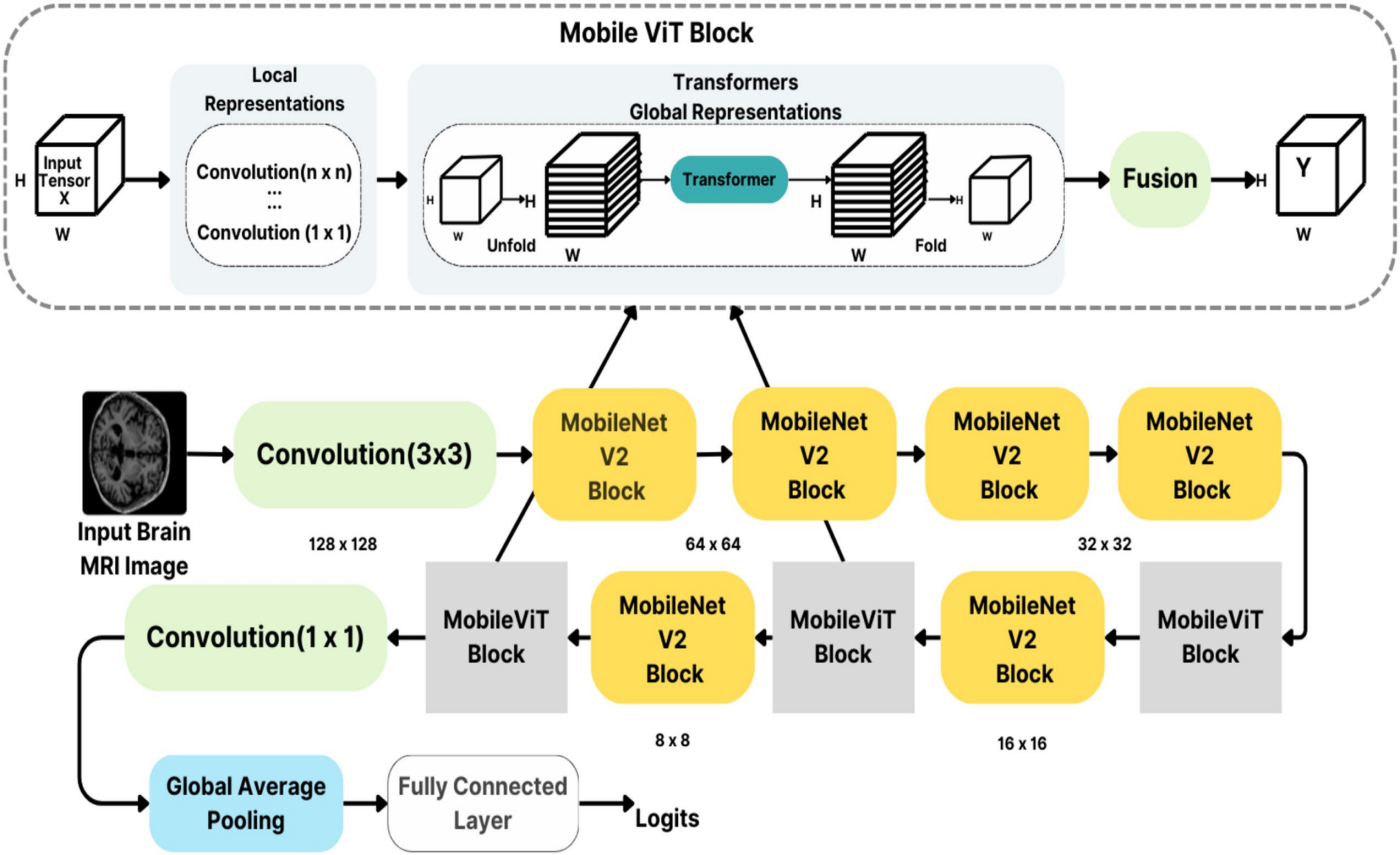
AlzheimerViT feature extraction and classification architecture based on MobileViT blocks.

MobileViT architecture, shown in Figure 13, starts with a 3×3 convolutional layer that down-samples the input image size from 256 × 256 to 128 × 128. Several subsequent MV2 blocks progressively down the sample spatially and expand the number of output channels. Each level will extract local features in this manner, increasing its output channels as its input image size decreases. After each down-sampling, the MobileViT blocks use transformers to model global dependencies across the patches, with the features processed in non-overlapping patches. This structure captures both local and global information efficiently, as the final feature map is passed through a global pooling layer and a fully connected layer to produce output for classification. Table 2 indicates a detailed breakdown of the layers, output sizes, strides, repeat counts, and output channels below:

**Table 2:** Detailed breakdown of AlzheimerViT architecture.

MobileViT Layers	Output Size	Output Stride	Repeat	Output Channels (S)
Image	256 × 256	1	-	3 (RGB)
Convolution(3×3) ↓ 2	128 × 128	2	1	16
MV2 Block 1	128 × 128	2	1	32
MV2 Block 2, ↓ 2	64 × 64	4	1	64
MV2 Block 3, ↓ 2	64 × 64	4	2	64
MV2 Block 4, ↓ 2	32 × 32	8	1	96
MobileViT Block (L = 2)	32 × 32	8	1	96 (*d* = 144)
MV2 Block 5, ↓ 2	16 × 16	16	1	128
MobileViT Block (L = 4)	16 × 16	16	1	128 (*d* = 192)
MV2 Block 6, ↓ 2	8 × 8	32	1	160
MobileViT Block (L = 3)	8 × 8	32	1	160 (*d* = 240)
Convolution(1×1)	8 × 8	32	1	640
Global Pooling	1 × 1	256	1	640
Linear (Fully Connected)	1 × 1	256	1	2 (output classes)

#### 3.4.1 Mathematical modelling

*Initial convolution*: The input MRI slice undergoes a 3×3 convolution with stride 2 to reduce its spatial dimensions and begin feature extraction, as shown in Equation 9.


(9) $$X\prime =\text{Convolution}(X,W_{\text{Convolution}},b_{\text{Convolution}})$$

#### 3.4.2 MobileViT blocks

a Expansion (1 × 1 Convolution)

The input is passed through a 1×1 convolution that increases the number of channels for the expansion of the feature space to richer representation, thereby allowing the model to capture more complex features before spatially reducing them as in Equation 10.


(10) $$X_{\text{expanded}}=\text{Convolution}(X\prime ,W_{\text{Expand}},b_{\text{Expand}})$$

b Depth-wise Convolution (3 × 3):

Each of the channels is separately convoluted with a 3×3 filter in the depth-wise manner that was explained in Equation 11. These operations do not have a high computational cost while maintaining spatial relationship-preserving within the individual channel, but they do decrease the parameters that would be required.


(11) $$X_{\text{depthwise}}=\text{Depthwise}(X_{\text{expanded}},W_{\text{depthwise}},b_{\text{depthwise}})$$

c Projection (1 × 1 Convolution):

The depth-wise convolution projects the set of channels from a large number to an even smaller set of channels by a 1×1 convolution as given by Equation 12. The result is a reduction in the size of the channel dimensions. It has three steps:


(12) $$X_{\text{Projected}}=\text{Convolution}(X_{\text{depthwise}},W_{\text{Project}},b_{\text{Project}}$$

*Pointwise Convolution*: It first increases the channel depth in a higher-dimensional space, as described by Equation 13, by a 1×1 convolution:


(13) $$X_{l}=\text{Convolution}(X_{L},X_{1\;x\;1,}b_{1\;x\;1})$$

##### 3.4.2.1 Global information encoding

The feature map unfolds into patches, which are then processed by transformer layers to capture long-range dependencies between patches. This is how the model is able to understand global structures in the MRI image, as Equation 14 shows.


(14) $$X_{U}=\text{Unfold}(X_{l})$$

##### 3.4.2.2 Concatenation and fusion

The local and global features (from depth-wise convolutions and transformers) are fused given in Equation 15, followed by the final convolution to integrate the same into a single tensor given by Equation 16. It’s then flattened for classification.


(15) $$\;X_{F}=\text{Fold}(X_{G})$$

(16) $$X_{\text{Concatenate}}=\text{Concatenate}(X_{L},X_{F})$$

##### 3.4.2.3 Final prediction

The flattened features are then used through a fully connected layer given by Equation 17 to predict whether the subject has Alzheimer’s, essentially a binary classification.


(17) $$X_{\text{Final}}=\text{Convolution}(X_{\text{Concatenate}},W_{\text{Final}},b_{\text{Final}})$$

#### 3.4.3 Pseudocode

**Table:** 

AlzheimerViT
1. *Input:* OASIS-3 Dataset
2. *Initial Convolution(3×3):* Perform 3 × 3 stride convolution, extract features$X\prime =\text{Convolution}(X,W_{\text{Convolution}},b_{\text{Convolution}})$
3. *AlzheimerViT Block* (Down Sampling and Feature Expansion) Expansion (1 × 1 convolution)$X_{\text{expanded}}=\text{Convolution}(X\prime ,W_{\text{Expand}},b_{\text{Expand}})$ Depth-wise Convolution (3 × 3):$X_{\text{depthwise}}=\text{Depthwise}(X_{\text{expanded}},W_{\text{depthwise}},b_{\text{depthwise}})$ Projection(1 × 1 convolution): $X_{\text{Projected}}=\text{Convolution}(X_{\text{depthwise}},W_{\text{Project}},b_{\text{Project}}$)
4. *Information Encoding (Local)*: Apply standard convolution and capture spatial information (Local) $X_{\text{Local}}=\text{Convolution}(X_{\text{Projected}},W_{\text{Local}},b_{\text{Local}})$
5. *Pointwise Convolution (Channel Projection)* Perform pointwise convolution and increase number of channels, also project the tensor to higher dimensional space. $X_{l}=\text{Convolution}(X_{L},X_{1\;x\;1,}b_{1\;x\;1})$
6. *Global Information Encoding (Transformer):* Unfold the higher dimension tensor from previous stage for global processing using transformer layers. $X_{U}=\text{Unfold}(X_{l})$
7. *Folding -Concatenation:* Succeeding to Transformer processing is folding operation of global information back to spatial dimensions. $X_{F}=\text{Fold}(X_{G})$ $X_{\text{Concatenate}}=\text{Concatenate}(X_{L},X_{F})$
8. *Final Pointwise Convolution:* Perform Final Pointwise Convolution, fuse the concatenated Features and decrease channel dimensionality. $X_{\text{Final}}=\text{Convolution}(X_{\text{Concatenate}},W_{\text{Final}},b_{\text{Final}})$
9. *Output Layer:* Add a Fully connected layer with an Activation (SoftMax) function Classes of Outputs, Alzheimer’s or Non-Alzheimer’s.
10. *Model Compilation* Specify Loss Function, an optimizer (possibly Adam, SGD) choose accuracy for the metric.
11. *Model Training* a. Feed into network the training samples in batch fashion. Backpropagate errors to update weights. b. Train and evaluate model on the validation data. If performance is not good enough, change the appropriate hyperparameters.
12. *Model Evaluation*: AlzheimerViT Model is trained and tested on OASIS-3 Dataset and performance is evaluated using Standard metrics like Accuracy, Specificity, Kappa Score.
13. *Model Usage:* Deploy the model for classification of unseen MRI brain scans into Alzheimer’s Disease categories with the predictions for clinical usage.

### 3.5 Hyperparameter tuning

Optimizing hyperparameters is a crucial step in enhancing deep learning models’ performance and generalization capability. For the AlzheimerViT model, we employed a systematic manual tuning strategy, refining hyperparameters through an iterative and empirical approach rather than automated search methods like grid search or random search, which are computationally expensive. The manual tuning approach focuses on targeted refinements, reducing unnecessary computations, allowing immediate adjustments and evaluation, and avoiding wasted trials on ineffective settings. Our manual tuning approach followed a structured process. Thus, we selected initial hyperparameter values based on best practices and previous research on vision transformers and MobileNetV2 architectures. Key metrics such as validation accuracy, loss behavior, and convergence stability were monitored at different training stages. Then, hyperparameters were gradually fine-tuned based on observed trends, including learning rate, batch size, label smoothing, and number of training epochs. Performance comparisons were made after each adjustment to assess improvements in classification accuracy and generalization. Therefore, the optimal set of hyperparameters was determined based on sustained improvements in validation accuracy and loss reduction over 400 + training epochs. Specifically, the final model was trained with a patch size of 4 (2 × 2), an image size of 256 × 256, an expansion factor of 4 for MobileNetV2 blocks, and a batch size of 64. The Adam optimizer was used with a learning rate of 1e-5. Furthermore, the categorical cross-entropy loss function with label smoothing (0.1) was incorporated to enhance generalization and mitigate overfitting. The final layers include a global pooling layer, followed by a fully connected (linear) output layer with two nodes corresponding to the binary classification task (Demented vs. Non-Demented). The output layer utilizes a softmax activation function, enabling probability-based predictions for each class. Training was conducted for over 400 epochs, continuously monitoring performance using validation accuracy as the primary evaluation metric. The best-performing model was automatically saved via the ModelCheckpoint callback, ensuring the optimal configuration was preserved.

## 4 Results and discussion

For the Alzheimer’s Disease prevention project, the experimental setup uses advanced computational resources, which include a CPU and an NVIDIA A100 GPU. The CPU with 256 MB of memory handles general tasks, such as data pre-processing and algorithm management, while the GPU with 40 GB of memory and compute capability 8.0 accelerates intensive parallel processing for deep learning models. The hardware setup has been chosen to process large amounts of medical data for efficient prediction and prevention of Alzheimer’s Disease through machine learning techniques because there is only one GPU available.

Key performance metrics are utilized to evaluate the AlzheimerViT model’s performance in predicting a person suffering from Alzheimer’s disease or healthy control. The confusion matrix provides a breakdown of true positives, false positives, true negatives, and false negatives, providing a visual perspective of model performance across all categories. Another common metric is the AUC-ROC curve, which explains the model’s ability to differentiate between output classes. A higher AUC curve indicates good performance and vice versa. The confusion matrix includes four major components: True Positives (TP), False Positives (FP), True Negatives (TN), and False Negatives (FN). These values are very important for the calculation of different performance metrics. For the case of Alzheimer’s Disease prediction using AlzheimerViT, True Positives (TP) refer to the accurate identification of Alzheimer’s Disease cases. At the same time, False Positives (FP) are healthy persons misclassified as having Alzheimer’s Disease. True negatives are healthy individuals who have been correctly classified, and false negatives represent cases of Alzheimer’s Disease wrongly classified as healthy.

Supplementary Figures S2–S4 provide a visualization of confusion Matrix plots obtained for Training, Validation, and testing the Oasis-3 dataset for predicting the Demented and Non-Demented classes, respectively.

**Figure 14:**
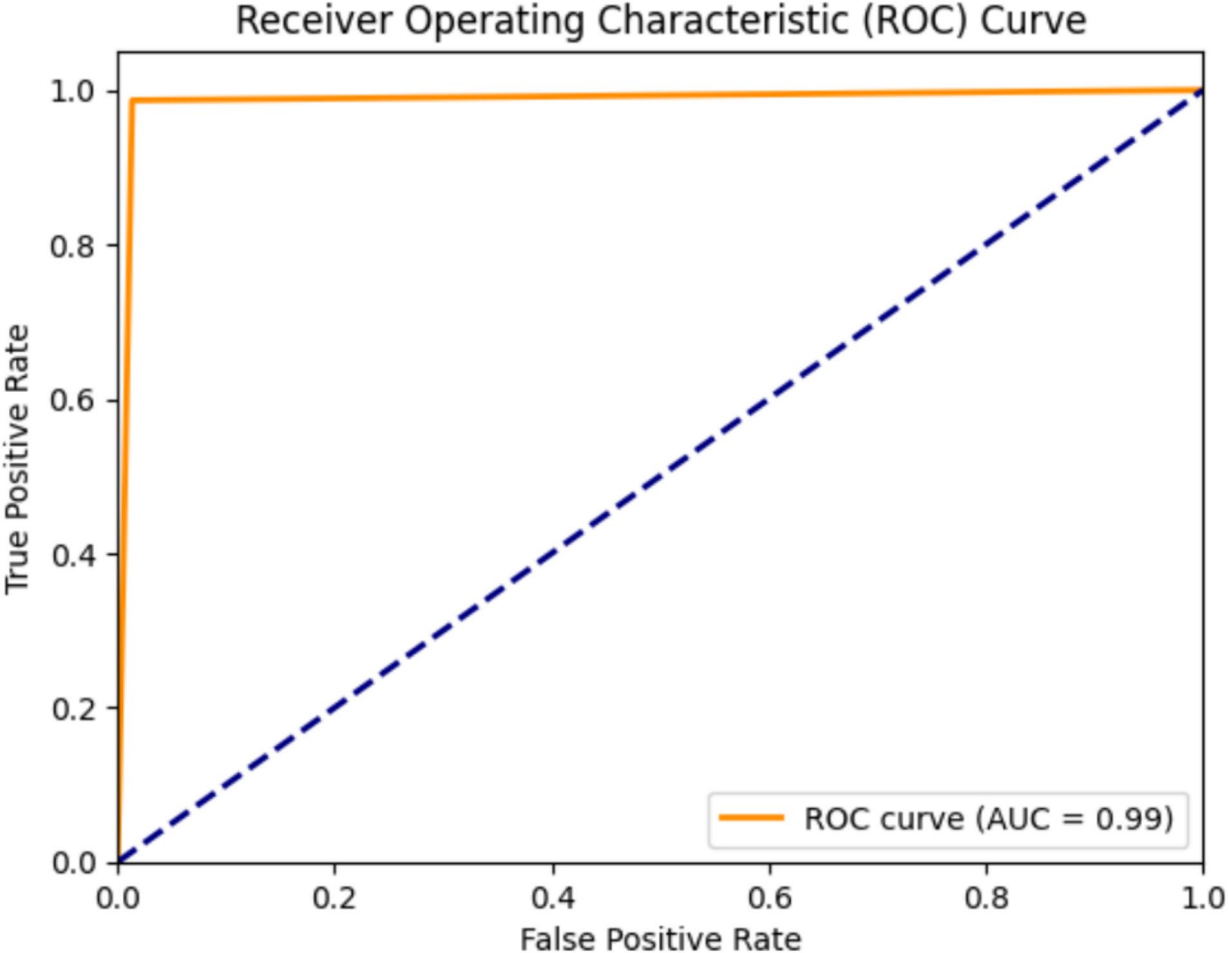
AUC-ROC plot for Alzheimer’s disease prediction using AlzheimerViT.

The AUC-ROC score for the proposed model is 0.99, indicating that the AlzheimerViT model performed highly in predicting Alzheimer’s using MRI images. Its high AUC score makes it reliable and robust, potentially making it a very useful tool for early detection, which will hopefully allow for more timely and accurate diagnoses in clinical settings. Figure 14 visually reconfirms this result, as shown by the ROC curve, proving that the model is excellent in discriminating between the two classes.

**Figure 15:**
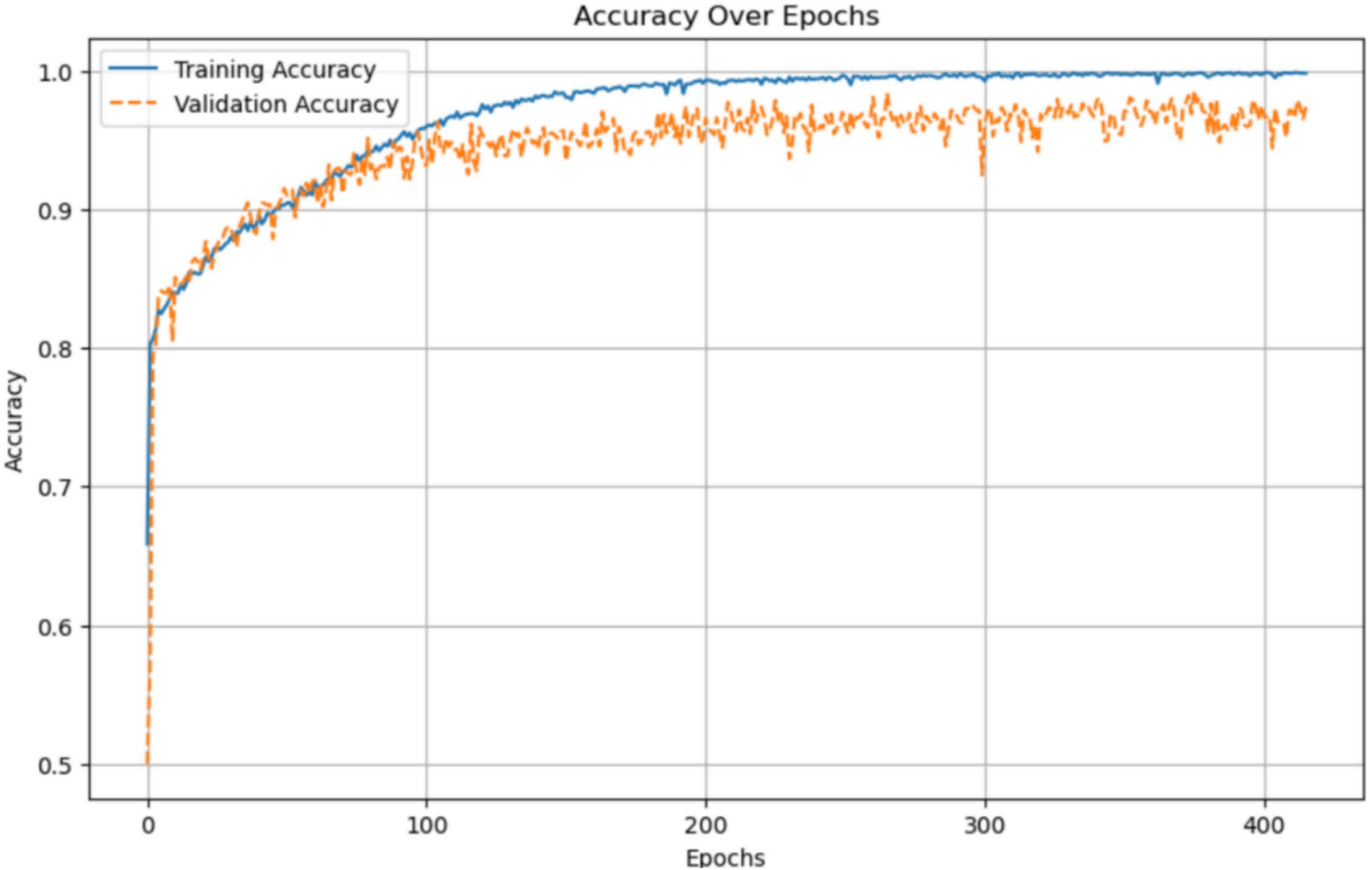
Accuracy per epoch plot for Alzheimer’s disease prediction using AlzheimerViT.

The Proposed Model was initially exposed to over 400 epochs of training and validation, as depicted in Figure 15. Training accuracy improves rapidly in the initial phase, reaching almost 100% accuracy in its final epochs, indicating the model’s effective learning capabilities. The validation accuracy fluctuates slightly but stabilizes at a high value, which suggests that the model generalizes reasonably well to unseen data while avoiding strong overfitting. Training and validation accuracy remain similar, which means that the training process is well-regularized.

**Figure 16:**
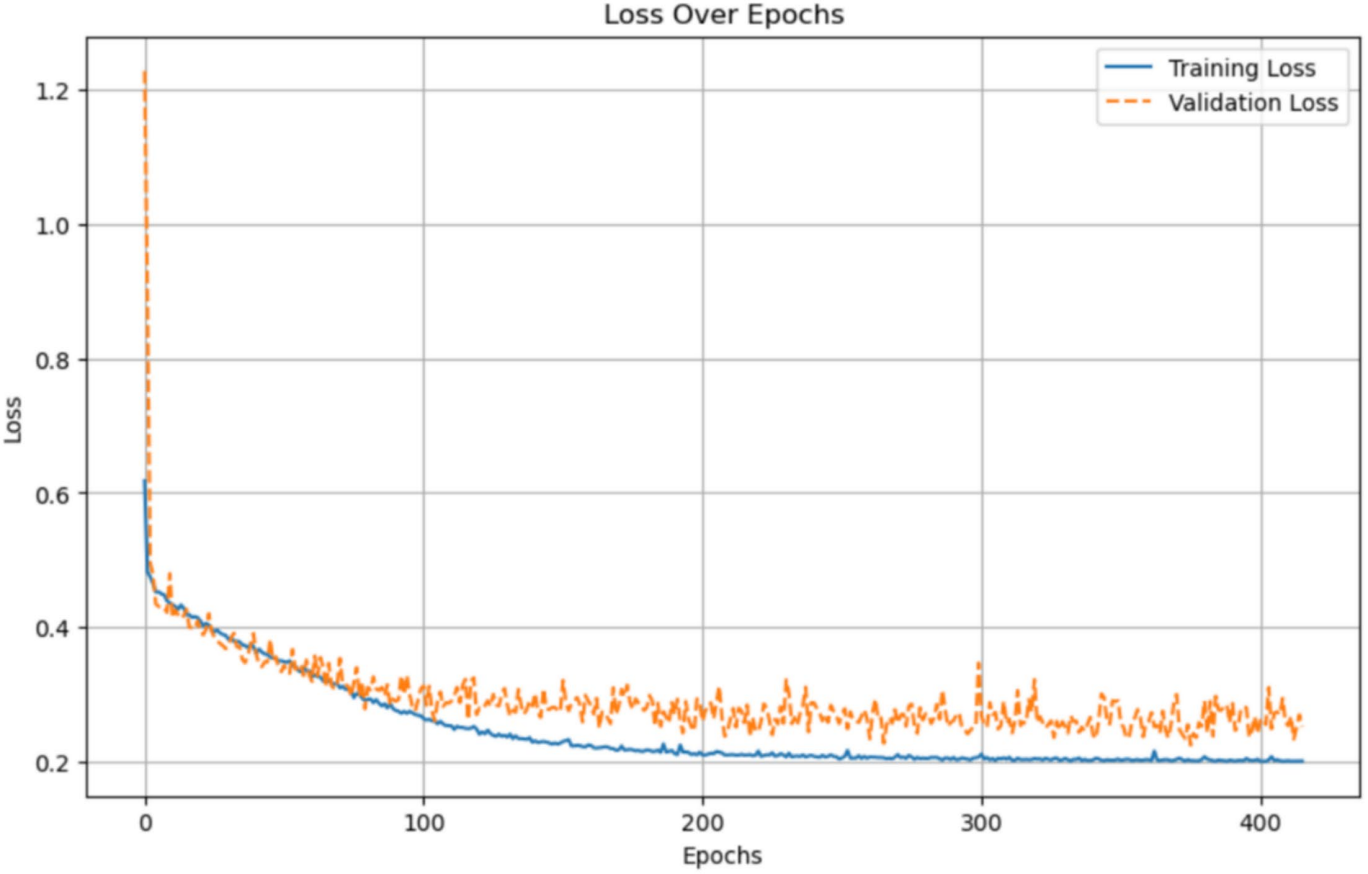
Loss per epoch plot for Alzheimer’s disease prediction using AlzheimerViT.

Figure 16 illustrates the training and validation loss of the AlzheimerViT model over 400 epochs. Both Training and Validation losses initially reduce very sharply, indicating that the model is learning to minimize errors well. Later, the training loss continues its reduction steadily and stabilizes at a low value, implying that the model fits the training data well. The validation loss also decreases but is less stable and has stabilized at a higher level than the training loss, indicative of some generalization variance. The small gap between the training and validation losses further indicates that the model will avoid strong overfitting and will perform well on data. A commonly used measure is overall accuracy, which is calculated as correctly classified instances over the total instances shown in Equation 18.


(18) $$\begin{array}{l} \text{Accuracy}=\\ \frac{\text{True Positives}+\text{True Negatives}}{\text{True Positives}+\text{True Negatives}+\text{False Positives}+\text{False negatives}} \end{array}$$

Table 3 presents the accuracy of different models for predicting Alzheimer’s disease, and the highest accuracy of 98.57% is obtained using the proposed AlzheimerViT model. Random Forest (95.53%), Support Vector Machine (95%), and XGBoost (92.30%) are other high-performing models. Gradient Boosted Machines (91.50%), Light Gradient Boosting (91.20%), and Logistic Regression (89%) also had strong performances. LSTM with Attention Mechanism (88.60%) and Relevance Vector Machine (88%) followed closely. Lower-performing models were Decision Tree with Ensemble Learning (87.2%), Extra Tree Classifier (85%), DRL-XGBoost (84.34%), Edge-Preservation Coherence Improvement algorithm (82%), Naive Bayes with feature selection (81%), and the default Naive Bayes (71.44%).

**Table 3:** Comparative analysis of accuracy of proposed AlzheimerViT and existing models for Alzheimer’s disease prediction.

Ref no.	Model name	Accuracy
**-**	AlzheimerViT (Proposed Model)	98.57%
(7)	Random Forest	95.53%
(11)	Support Vector Machine	95%
(33)	XG-Boost Model	92.30%
(32)	Gradient Boosted Machines (GBM)	91.50%
(10)	Light Gradient Boost.	91.20%
(31)	LSTM with Attention Mechanism	88.60%
(34)	Relevance Vector Machine (RVM)	88%
(16)	Decision Tree with Ensemble Learning	87.20%
(29)	Extra Tree Classifier	85%
(13)	DRL-XGBOOST	84.34%
(30)	EP-CI algorithm	82%
(28)	Naive Bayes	81%
(9)	Logistic regression model	89.00%
(12)	Naive Bayes	71.44%

Error rate is an essential metric in evaluating a model’s performance. The error rate provides information on the number of incorrect predictions or decisions made by the model, as explained in Equation 19.


(19) Error Rate=100−Accuracy%

Table 4 gives the error rates of different machine learning models, ranging from extremely low to comparatively high. The AlzheimerViT (Proposed Model) has a minimum error rate of 1.43%, followed by Random Forest (4.47%) and Support Vector Machine (5%). Some other models, like XG-Boost, Gradient Boosted Machines, and Light Gradient Boost, have error rates of 7.7, 8.5, and 8.8%, respectively. Models based on LSTM with Attention Mechanism and Relevance Vector Machine have 11.4 and 12% error rates. In contrast, the Decision Tree with Ensemble Learning and Extra Tree Classifier have higher error rates of 12.8 and 15%. DRL-XGBOOST, EP-CI algorithm (Image enhancement), and Naive Bayes classifier with feature selection methods have error rates of 15.66, 18, and 19%, respectively. The logistic regression model has an 11% error rate, and Naive Bayes has a maximum error rate of 28.56%.

**Table 4:** Comparative analysis of error-rate of proposed and existing models for Alzheimer’s disease prediction.

Ref No.	Model	Error rate (%)
-	AlzheimerViT (Proposed Model)	1.43%
(7)	Random Forest	4.47**%**
(11)	Support Vector Machine	5**%**
(33)	XG-Boost Model	7.7**%**
(32)	Gradient Boosted Machines (GBM)	8.5**%**
(10)	Light Gradient Boost.	8.8**%**
(31)	LSTM with Attention Mechanism	11.4**%**
(34)	Relevance Vector Machine (RVM)	12**%**
(16)	Decision Tree with Ensemble Learning	12.8**%**
(29)	Extra Tree Classifier	15**%**
(13)	DRL-XGBOOST	15.66**%**
(30)	EP-CI algorithm	18**%**
(28)	Naive Bayes	19**%**
(9)	Logistic regression model	11**%**
(12)	Naive Bayes	28.56**%**

Accuracy may not always be enough, especially with imbalanced datasets, which necessitates the calculation of precision and Recall. Precision is the fraction of actual positive predictions out of all instances labeled positive, given in Equation 20, while Recall or sensitivity measures the fraction of correctly predicted true positives out of all actual cases that were indeed positive, as explained in Equation 21. Table 5 is a comparative analysis of the Precision % of the proposed AlzheimerViT Alzheimer’s disease prediction model and existing Methodologies.


(20) $$\text{Precision}=\frac{\text{True Positives}}{\text{True Positives}+\text{False Positives}}$$

(21) $$\text{Recall}=\frac{\text{True Positives}}{\text{True Positives}+\text{False Negatives}}$$

**Table 5:** Comparative analysis of precision of proposed and existing models for Alzheimer’s disease prediction.

Algorithm	Precision (%)
AlzheimerViT (Proposed System)	98.7
SVM (29)	77
Voting Classifier (29)	83
Logistic Regression (29)	74.7
XGBoost (29)	85
Decision Tree Classifier (29)	80
RF Classifier (29)	85

The recall performance of various machine learning models for the prediction of Alzheimer’s disease is shown in Table 6. Comparative analysis of Recall of Proposed and Existing Models for Alzheimer’s Disease Prediction. Obviously, the proposed system is the AlzheimerViT, which had a remarkable recall of 98.47% due to its sensitivity for the detection of cases of Alzheimer’s disease. Decision Tree Classifier, XG-Boost, and Voting Classifier recall values were in the 79 to 83% range; the Ensemble methods with feature selection achieved a recall value of 80% each: AD-CT and AD-MCI. By contrast, the Ensemble with feature selection had the lowest Recall, 50%, which is MCI-CT. The other two models - Logistic Regression and Random Forest - give recall values of 70%. It can be considered a mediocre performance.

**Table 6:** Comparative analysis of recall of proposed and existing models for Alzheimer’s disease prediction.

Algorithm	Recall (%)
AlzheimerViT (Proposed System)	98.47
Decision Tree Classifier (29)	79
XGBoost (29)	80
Voting Classifier (29)	83
Logistic Regression (15)	70
Random Forest (15)	70
Ensemble with feature selection (AD-CT) (16)	80
Ensemble with feature selection (AD-MCI) (16)	80
Ensemble with feature selection (MCI-CT) (16)	50

Sensitivity is important because it needs to identify most patients who have Alzheimer’s Disease in order not to miss most of those. Specificity measures how well the model is performing in getting the right positives, that is, not the people without Alzheimer’s Disease, true negatives explained in Equation 22. It shows that the model is not committing false positives. These metrics, all put together, provide a complete evaluation of reliability and robustness in its predictions for the AlzheimerViT model in terms of minimizing errors like false positives and negatives and ensuring good performance on its predictions of positive and negative cases.


(22) $$\text{Specificity}=\frac{\text{True Negatives}}{\text{True Negatives}+\text{False Positives}}$$

The data in Table 7. Comparative Analysis of Specificity % of Proposed and Existing Models for Alzheimer’s Disease Prediction compares the specificity performance of different machine learning models in detecting Alzheimer’s disease. The proposed system, AlzheimerViT, has the highest specificity at 98.67%, meaning it is very effective at correctly identifying negative cases (non-Alzheimer’s). Other models are ICAE (Transfer Learning) and ICAE, with specificity values of 70.71 and 60.41%, respectively. Ensemble methods with feature selection are AD-MCI and AD-CT, which have the same specificity value of 67%. SVM with RBF is moderate, with a specificity equal to 87.17%, while CAE and CAE (Transfer Learning) specificity scores are 60.04 and 71.53%, respectively. The Feature Selection Model with the Ensemble is known as MCI-CT, which has the lowest specificity at 43%. Another noteworthy point was that the qEEG Processing Technique has a specificity value of 91.7.

**Table 7:** Comparative analysis of specificity % of proposed and existing models for Alzheimer’s disease prediction.

Algorithm	Specificity (%)
AlzheimerViT (Proposed System)	98.67
ICAE (Transfer Learning) (36)	70.71
ICAE (36)	60.41
Ensemble with feature selection (AD-MCI) (16)	67
SVM with RBF (17)	87.17
CAE (36)	60.04
Ensemble with feature selection (MCI-CT) (16)	43
Ensemble with feature selection (AD-CT) (16)	67
Multilayer Perceptron (18)	79.4
LIBS-ML (19)	75
CAE (Transfer Learning) (36)	71.53
qEEG Processing Technique (20)	91.7

Table 8 provides the Comparative analysis of the Kappa score of proposed and existing models for Alzheimer’s Disease Prediction. It is evident that the proposed system, AlzheimerViT, has performed the best among all other machine learning algorithms to classify Alzheimer’s disease. It has a maximum Kappa score of 97.2%. In comparison with other models, it outperforms others like Naïve Bayes with 54% and SVM with 54%. In contrast, methods like Random Forest + PSO (88%) and C4.5 + PSO (82%) also show strong results, though they still lag behind the proposed system.

**Table 8:** Comparative analysis of Kappa score of proposed and existing models for Alzheimer’s disease prediction.

Algorithm	Kappa score (%)
AlzheimerViT (Proposed System)	97.2
Naïve Bayes	54
Logistic regression	78
SVM	54
Random forest	84
C4.5	79
CHAID	80
ID3	80
C4.5 + PSO	82
Random forest + PSO	88
ID3 + PSO	80.8

We conducted inference time and classification performance tests on the AlzheimerViT system. Our evaluation study aimed to evaluate the effectiveness of AlzheimerViT in classifying images and determining inference time results on resource-constrained devices. Our experimental setup measured AlzheimerViT’s inference time with CPU and GPU environments. A batch of 64 images was used for the GPU, while one image per batch was used for an 8-core CPU. The inference time test results are presented in Table 9. AlzheimerViT, with 7 M parameters and 81.3 MB size, can evaluate within 410 ms for the typical batch size of 64 images with GPU and within 11 ms for a single batch with CPU. The results have important implications for developing and deploying the AlzheimerViT system to predict Alzheimer’s disease in practical resource-constrained edge environments.

**Table 9:** AlzheimerViT inference time on CPU and GPU.

Algorithm	Model size (MB)	Total parameters	Inference Time (ms)	
			CPU batch size (1 image)	GPU batch size (64 images)
AlzheimerViT (Proposed System)	81.3 MB	7,039,714	11 ms	410 ms

Grad-CAM is a visualization technique that enhances the interpretability of deep learning models by highlighting the regions of input images that significantly influence the model’s predictions. In the context of Alzheimer’s disease detection, Grad-CAM provides valuable insights into the specific areas of brain MRI scans that the model considers when classifying the disease. The Grad-CAM heatmaps for the Non-Demented class, as shown in Supplementary Figure S5, exhibit relatively lower activation intensities compared to the Demented class depicted in Supplementary Figure S6. The activations in the Non-Demented class are dispersed and do not concentrate on distinct regions of the brain. This pattern suggests that the model associates the overall structural integrity of the brain with the non-demented classification, reflecting the absence of significant pathological changes. In contrast, the heatmaps for the Demented class in Supplementary Figure S6 display strong and focused activations in specific brain regions such as the hippocampal region, entorhinal cortex, and parietal and temporal lobes. The vibrant colours and concentrated patterns indicate the high importance of these regions in the model’s decision-making process. Supplementary Figures S5, S6 illustrate the interpretability of the AlzheimerViT system, offering a clear and intuitive understanding of its decision-making process. It enhances the model’s transparency and reliability, making it a valuable tool for clinicians and researchers seeking to understand and validate AI-driven diagnostic decisions.

## 5 Conclusion

The proposed system utilizes AlzheimerViT, a light and efficient vision transformer, to make early detection and classification using MRI images from the OASIS-3 dataset on Alzheimer’s disease. A good performance of 98.57% in terms of accuracy, precision at 98.7%, and recall at 98.47% with a specificity of 98.67% and a Kappa score of 97.2%, AUC-ROC Score of 0.99 further points toward its suitability for application within the clinical setting in deriving reliable and accurate predictions. Despite its excellent performance, the model does have limitations and includes reliance on high-quality data, possible overfitting, and sufficient samples for generalizing, the “black box” characteristic of deep-learning models making it difficult to interpret their decision-making mechanism.

Future work may be the improvement of the model’s robustness and handling of possible overfitting by using more diverse and even more extensive datasets and applying transfer learning to fine-tune the model. Even though Grad-CAM was employed to improve interpretability, future work can be based on other methods to explain the model predictions and avoid misrepresenting visual information and over-reliance on AI. In addition, integrating other complementary sources of data, such as genetic information or longitudinal MRI scans, may improve the model’s predictive capabilities, providing an all-inclusive tool for the early diagnosis of Alzheimer’s and personalized treatment planning.

## Data Availability

The original contributions presented in the study are included in the article/Supplementary material, further inquiries can be directed to the corresponding author.

## Glossary

OASIS: Open Access series of Imaging Studies
MRI: Magnetic Resonance Imaging
ADNI: Alzheimer’s Disease Neuroimaging Initiative
AUC-ROC: Area under the Receiver operating characteristics curve
AD: Alzheimer’s Disease
DenseNet201: Densely connected convolutional network
RESNET: Residual Network
Grad-Cam: Gradient weighted class activation mapping
MCI: Mild Cognitive Impairment
EMCI: early mild cognitive impairment
MLP: Multilayer Perceptron
MobileViT: Mobile Vision Transformer
CAE: Convolutional Auto Encoder
CHAID: Chi-Squared Automatic Interaction Detection
SVM: Support Vector Machine
RF: Random Forest Classifier
PSO: Particle Swarm Optimization
ICAE: Improved convolutional Auto-encoder
AD-CT: Alzheimer’s Disease cognitive testing
AD-MCI: Alzheimer’s Disease Mild Cognitive impairment
MCI-CT: Mild cognitive impairment cognitive testing
M-CapNets: Modified Capsule Networks
VGG: Visual Geometry
EDCM: Ensemble decision curve modeling
