# Supplementary figures and images for: AlzheimerViT: harnessing lightweight vision transformer architecture for proactive Alzheimer’s screening
Source: Front Med (Lausanne). 2025 Jun 17;12:1568312. doi: 10.3389/fmed.2025.1568312 (PMC12210019; doi:10.3389/fmed.2025.1568312)

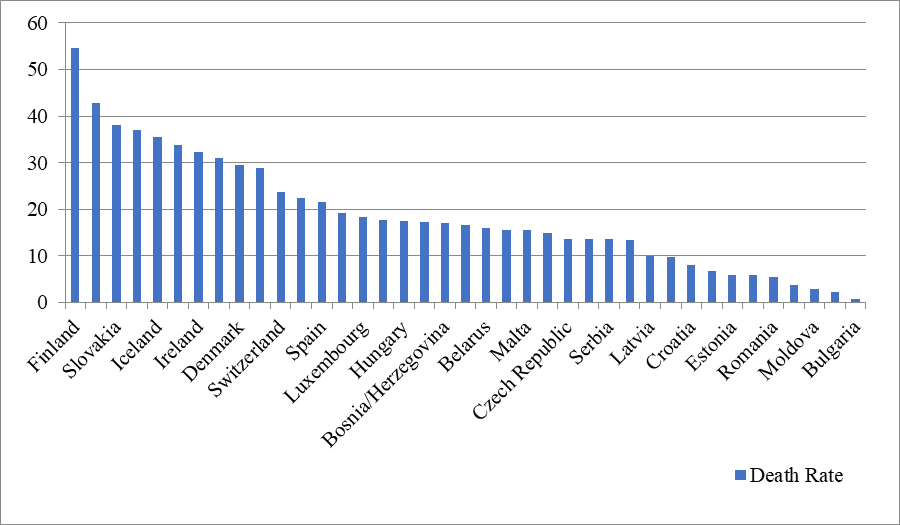

Supplement: Supplementary file 1 [file Supplementary_file_1.zip › Supplementary file 1/Alzheimer's_Figures/Figure 1.png]

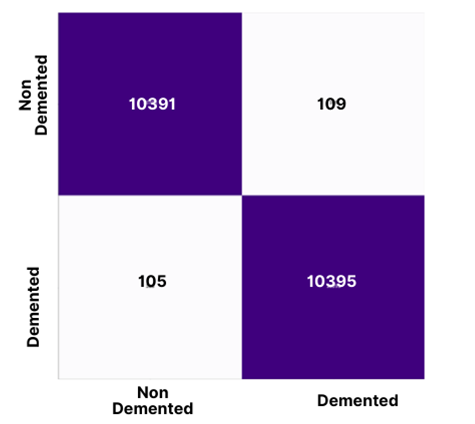

Supplement: Supplementary file 1 [file Supplementary_file_1.zip › Supplementary file 1/Alzheimer's_Figures/Figure 2.png]

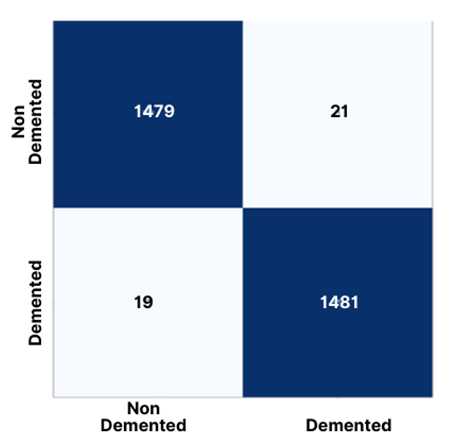

Supplement: Supplementary file 1 [file Supplementary_file_1.zip › Supplementary file 1/Alzheimer's_Figures/Figure 3.png]

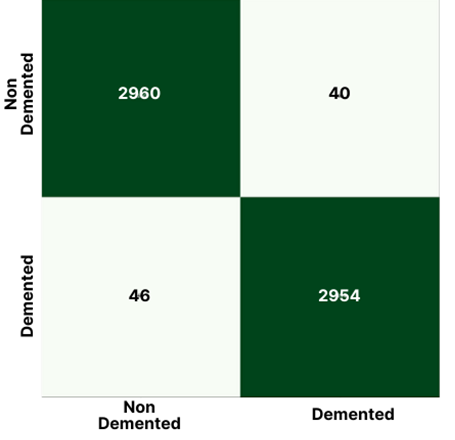

Supplement: Supplementary file 1 [file Supplementary_file_1.zip › Supplementary file 1/Alzheimer's_Figures/Figure 4.png]

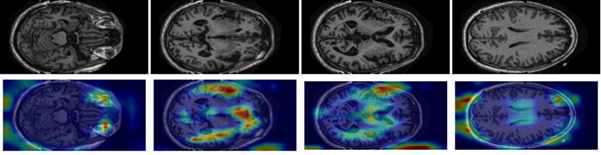

Supplement: Supplementary file 1 [file Supplementary_file_1.zip › Supplementary file 1/Alzheimer's_Figures/Figure 5.png]

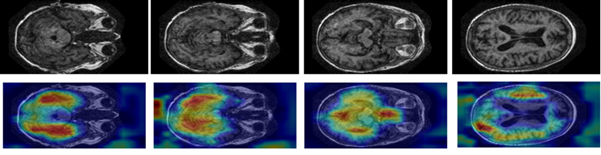

Supplement: Supplementary file 1 [file Supplementary_file_1.zip › Supplementary file 1/Alzheimer's_Figures/Figure 6.png]
